# Biologics and the psoriatic march: Is it time to rethink early intervention?

**DOI:** 10.1111/jdv.70394

**Published:** 2026-04-25

**Authors:** Paolo Gisondi

**Affiliations:** ^1^ Section of Dermatology and Venereology, Department of Medicine University of Verona Verona Italy

The study by Miao et al. provides an important contribution to the ongoing debate regarding whether biologic therapies for psoriasis may reduce the risk of developing psoriatic arthritis (PsA).^1^ Using a large US administrative claims database and a rigorous methodological approach, the authors report that patients with moderate‐to‐severe psoriasis who transitioned from phototherapy to biologic therapy experienced a significantly lower incidence of PsA than those who continued phototherapy alone. This finding supports the hypothesis that biologics may exert a protective effect on the evolution from cutaneous to musculoskeletal disease (Table 1).^2^, ^3^, ^4^, ^5^

**TABLE 1:** Studies reporting that biologic therapy for PsO can reduce the risk of PsA development compared to a control group.

First author	Sample size	Comparator	Relative risk reduction
Solmaz D	203	Topical therapy	68%
Gisondi P	464	Phototherapy (nb‐UVB)	73%
Floris A	1023	Topicals, phototherapy, conventional systemic drugs	77%
Acosta Felquer ML	1719	Topicals / no treatment / phototherapy	81%
Piaserico S	946	Phototherapy	68%
Miao KL	26,470	Phototherapy	34%

A major strength of the study lies in its thoughtful design, which addresses two key sources of bias that have affected previous analyses: protopathic bias and confounding‐by‐indication. By excluding PsA cases diagnosed within 1 year of biologic initiation, the authors reduce the likelihood that early, subclinical joint symptoms prompted the switch to biologics. Similarly, by restricting the baseline population to patients who had all received phototherapy—used here as a proxy for moderate‐to‐severe psoriasis—the study ensures a more comparable starting point between cohorts. This is a notable improvement over earlier claim‐based studies that compared biologic users with patients treated only with topicals or mild systemic agents, inadvertently mixing populations with very different baseline risks.

The results are compelling: The fully adjusted hazard ratio for PsA development among biologic users was 0.66, indicating a 34% relative risk reduction. This effect persisted across multiple sensitivity analyses, including age‐, sex‐ and ethnicity‐matched cohorts. Although observational data cannot establish causality, the consistency of the findings strengthens the argument that biologics may modify the natural history of psoriatic disease.

Biologically, this hypothesis is plausible. Psoriasis and PsA share overlapping immunopathogenic pathways, particularly involving TNF‐α, IL‐17 and IL‐23. Subclinical entheseal inflammation is well‐documented in psoriasis patients, even in the absence of joint symptoms. Early suppression of these inflammatory pathways may theoretically prevent progression to clinical PsA. Indeed, imaging studies have shown that biologics can reduce enthesitis and synovitis detectable by ultrasound or MRI, even in patients without established PsA.

However, several limitations warrant consideration. First, the use of phototherapy as a surrogate for disease severity, while pragmatic, is imperfect; treatment selection is influenced by patient preference, access and physician practice patterns. Second, claims databases lack granular clinical data such as PASI scores, body surface area, nail involvement or family history—factors known to influence PsA risk. Third, although PsA diagnoses were restricted to rheumatologists, misclassification remains possible. Finally, the study does not differentiate between biologic classes; whether IL‐23 inhibitors, IL‐17 inhibitors, or TNF inhibitors differ in their preventive potential remains debatable.

Despite these limitations, the study adds weight to a growing body of evidence suggesting that early systemic intervention may alter the trajectory of psoriatic disease.^1^, ^2^, ^3^, ^4^, ^5^ If confirmed in prospective studies, this could have meaningful implications for clinical practice. Dermatologists may increasingly consider early biologic therapy not only to control skin inflammation but also to reduce the long‐term burden of PsA, a condition associated with irreversible joint damage, disability and reduced quality of life.

Future research should focus on prospective cohorts with standardized clinical assessments, imaging biomarkers and stratification by biologic class. Randomized controlled trials designed specifically to evaluate PsA prevention—although challenging—would provide the highest level of evidence. Until then, the findings by Miao et al. represent an important step towards understanding how timely intervention may reshape the natural history of psoriatic disease.

## CONFLICT OF INTEREST STATEMENT

Gisondi P. has received honoraria for service as consultant and/or speaker for AbbVie, Almirall, Bristol‐Myers Squibb, Eli Lilly, LEO Pharma, Johnson & Johnson, Novartis, Pfizer, Takeda, and UCB.

## Data Availability

Data sharing not applicable to this article as no datasets were generated or analysed during the current study.
